# Solubilization of Phospholipid by Surfactin Leading to Lipid Nanodisc and Fibrous Architecture Formation

**DOI:** 10.3390/molecules29061300

**Published:** 2024-03-14

**Authors:** Tomohiro Imura, Satohiro Yanagisawa, Yuri Ikeda, Ryodai Moriyama, Kenichi Sakai, Hideki Sakai, Toshiaki Taira

**Affiliations:** 1 Research Institute for Chemical Process Technology, National Institute of Advanced Industrial Science and Technology (AIST), 4-2-1 Nigatake, Miyagino, Sendai 983-8551, Japan; 2 New Business Development Division, Kaneka Corporation, 2-3-18, Nakanoshima, Kita-ku, Osaka 530-8288, Japan; satohiro.yanagisawa@kaneka.co.jp; 3 Faculty of Science and Technology, Tokyo University of Science, 2641 Yamazaki, Noda 278-8510, Japank-sakai@rs.tus.ac.jp (K.S.); hisakai@rs.tus.ac.jp (H.S.)

**Keywords:** surfactin, phospholipid, nanodiscs, fibrous aggregates

## Abstract

Nanodiscs belong to a category of water-soluble lipid bilayer nanoparticles. In vivo nanodisc platforms are useful for studying isolated membrane proteins in their native lipid environment. Thus, the development of a practical method for nanodisc reconstruction has garnered consider-able research interest. This paper reports the self-assembly of a mixture of bio-derived cyclic peptide, surfactin (SF), and l-α-dimyristoylphosphatidylcholine (DMPC). We found that SF induced the solubilization of DMPC multilamellar vesicles to form their nanodiscs, which was confirmed by size-exclusion chromatography, dynamic light scattering, and transmission electron microscopy analyses. Owing to its amphiphilic nature, the self-assembled structure prevents the exposure of the hydrophobic lipid core to aqueous media, thus embedding ubiquinol (CoQ10) as a hydrophobic model compound within the inner region of the nanodiscs. These results highlight the feasibility of preparing nanodiscs without the need for laborious procedures, thereby showcasing their potential to serve as promising carriers for membrane proteins and various organic compounds. Additionally, the regulated self-assembly of the DMPC/SF mixture led to the formation of fibrous architectures. These results show the potential of this mixture to function as a nanoscale membrane surface for investigating molecular recognition events.

## 1. Introduction

The self-assembly of phospholipids into various lamellar structures is an essential natural phenomenon [1]. Biological membranes comprise different types of phospholipids formed via self-assembly, which form natural borders for metabolic substances and signal molecules [2]. Membrane proteins exclusively facilitate the translocation of molecules and signals across cellular membranes. These proteins perform numerous key functions in cellular metabolism and signalling, constituting approximately 30% of the human proteome, representing the target of choice for more than half of all presently marketed drugs [3,4,5,6].

Nanodiscs belong to a category of water-soluble lipid bilayer nanoparticles [7,8,9]. In vivo nanodisc platforms are useful for studying isolated membrane proteins in their native lipid environment [10]. Thus, the development of a practical method for nanodisc reconstruction has garnered considerable research interest. Recent studies have reported the self-assembly of nanodiscs through biomimetic helical peptides [11,12,13,14,15]. This technique involves the spontaneous structural conversion of phospholipid vesicles (liposomes) into nanodiscs through the straightforward addition of amphiphilic peptides. These amphiphilic helical peptides function as a surfactant, adeptly solubilizing the phospholipid bilayer while stabilizing the edge of the bilayer’s rim [16,17,18]. Owing to their amphiphilic nature, these self-assembled structures prevent the exposure of the hydrophobic lipid core to aqueous media, thus providing relative stability and monodispersity. These nanodiscs are potentially useful over conventional surfactant micelles and liposomes that can be applied to the incorporation of biomolecules, not only proteins but also bioactive compounds. However, despite the apparent simplicity of this procedure, it relies on synthesizing amphiphilic helical peptides, which is cumbersome and does not generate sufficient yield. Surfactin (SF) is a bio-derived amphiphilic peptide abundantly produced from renewable resources by Bacillus subtilis [19,20,21]. SF exhibits high biodegradability, in addition to high surface activity and unique self-assembling properties. It is also known that SF interacts with lipid bilayers constituting cellular membranes. In this study, we report the formation of nanodiscs without using any synthetic peptides. We found that adding SF to l-α-dimyristoylphosphatidylcholine (DMPC) multilamellar vesicles (MLVs) prompts the formation of nanodiscs. Owing to the hydrophobic lipid core, the nanodiscs can incorporate the hydrophobic lipid ubiquinol (CoQ10). At high concentrations, the mixture of SF and DMPC also produced fibrous nanostructures which entangled with each other to yield highly viscous aqueous solutions. 

## 2. Results

### 2.1. Ability of SF to Solubilize DMPC

**Figure 1:**
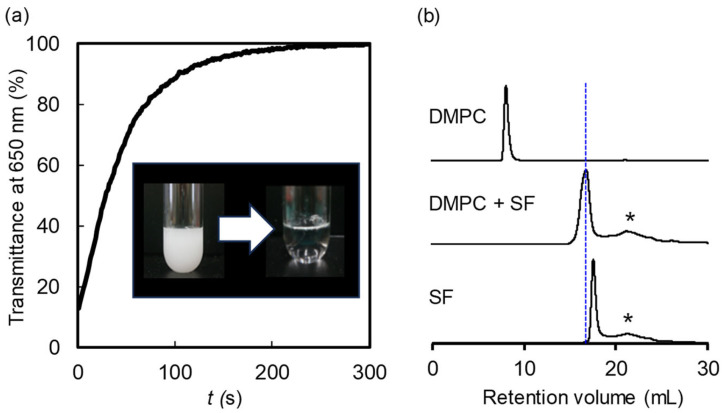
(**a**) Transmittance measurement of the mixture of DMPC and SF at 650 nm. The inset shows the visual observation before and after adding SF to DMPC MLVs. (**b**) SEC profiles of DMPC (top), the mixture of SF and DMPC (middle), and SF (bottom). The Stokes diameter of the nanoparticles was determined by comparing their SEC retention volume to standard curves of four globular proteins. Asterisks represent impurities in SF sample.

First, we investigated the effect of SF on the solubilization of phospholipids. Our approach involved the preparation of DMPC multilamellar vesicles (MLVs) within phosphate-buffered saline (PBS) [16]. The aqueous suspension of MLVs (1 mM) exhibited turbidity. Subsequently, SF was introduced to the turbid solution in a 1:1 DMPC:SF molar ratio. By maintaining the transmittance at 650 nm, we found that within 240 s, SF induced the solubilization of the MLVs to form a transparent solution (Figure 1a). This result clearly revealed the distinct ability of SF to solubilize DMPC. The size-exclusion chromatography (SEC) analysis of DMPC displayed a peak at 8.3 mL, which is the void volume originating from the MLVs, while the addition of SF to the MLVs yielded a peak at 16.8 mL with a Stokes diameter of 8.5 nm (Figure 1b). The diameter of the SF and DMPC mixture exceeded that of the SF micelle (the elution curve displayed a peak at 17.5 mL), corresponding to an estimated Stokes diameter of 6.8 nm. Notably, the Stokes diameter of the DMPC/SF mixture was increased with increasing DMPC molar ratio (8.8 nm at DMPC/SF = 2/1, 10.6 nm at DMPC/SF = 3/1, and 11.7 nm at DMPC/SF = 4/1). These results elucidate the formation of a lipid–peptide complex from DMPC and SF, leading to the formation of nano-sized aggregates.

### 2.2. Nanodisc Formation

**Figure 2:**
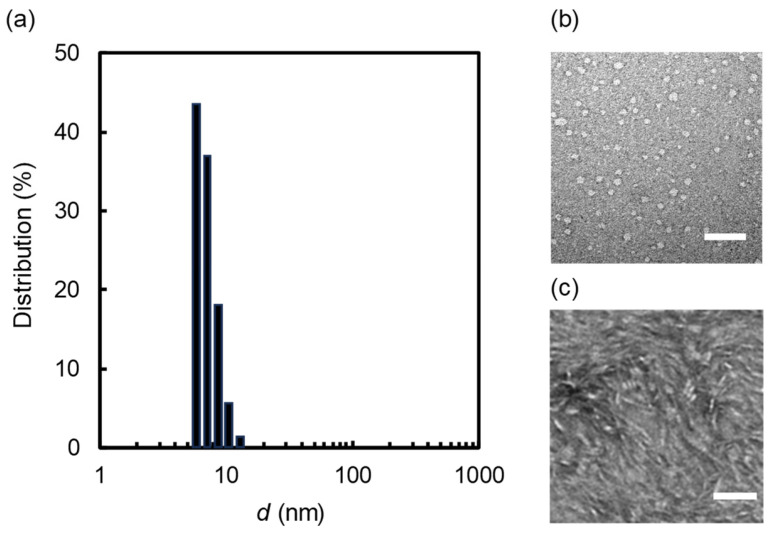
(**a**) DLS size distribution of the nanoparticles prepared from SF (1 mM) and DMPC (1 mM). NS-TEM images of the nanoparticles prepared from SF (1 mM) and DMPC (1 mM) (scale bar = 50 nm). (**b**) Uranyl acetate was used as the stain. (**c**) Phosphor tungstic acid was used as the stain.

The dynamic light scattering (DLS) measurement of the transparent solution (SF = DMPC = 1 mM) revealed a notably narrow size distribution, with an average diameter of 6.8 ± 1.4 nm, as shown in Figure 2a. Notably, the average diameter aligned closely with the SEC analysis (Figure 1b). To confirm the morphology of the nano-sized aggregates formed from the mixture of DMPC and SF, a negative-stain transmission electron microscopy (NS-TEM) analysis was performed. The captured images of the aliquots depicted both circular (Figure 2b) and rectangular objects (Figure 2c). The difference in morphology between the images was probably caused by the NS-TEM protocol. In the case of Figure 2b, uranyl acetate was used as the stain, while phosphor tungstic acid was used in the case of Figure 2c. The size of the circular objects (7.3 ± 1.6 nm) is comparable to those obtained from the DLS and SEC analyses. The thickness of the rectangular objects roughly estimated by the NS-TEM image was 4.8 ± 0.9 nm, which corresponds well to the thickness of the DMPC bilayers (ca. 5.1 nm) constructing nanodiscs with membrane scaffold proteins [22]. This result partially supports the beltlike model of nanodisc structure. Notably, some rectangular objects were stacked into a rouleaux formation in the image. These NS-TEM images consistently paralleled those typically associated with nanodiscs where the phospholipid bilayer membranes are stabilized by membrane scaffold proteins [7], peptides [16,17,18], or polymers [23]. 

**Figure 3:**
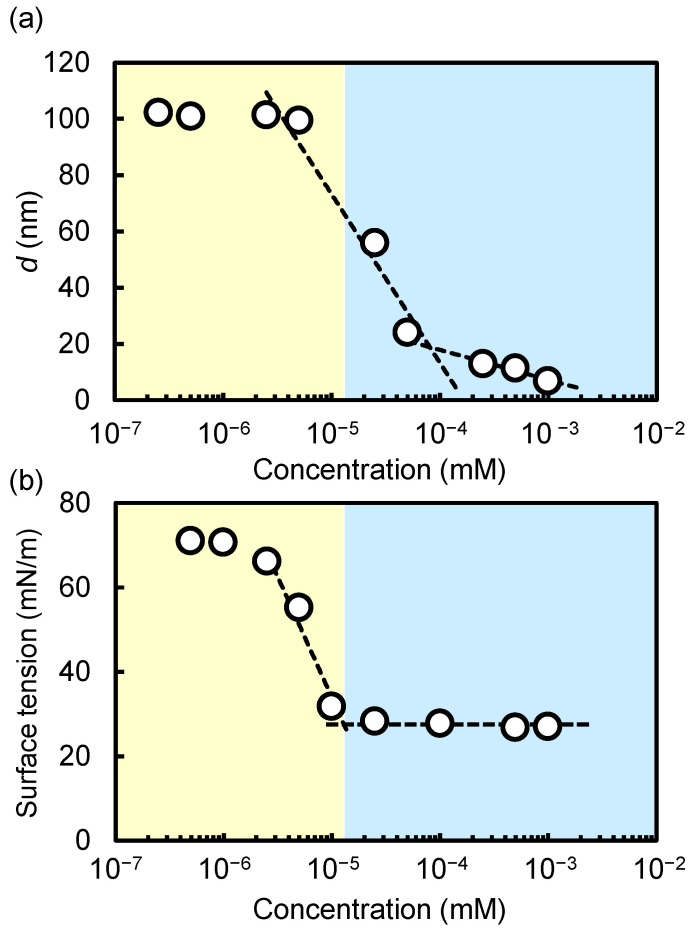
(**a**) Relationship between DLS size distribution and concentration of SF/DMPC (=1/1) mixture in PBS buffer (pH 7.4, 25 °C). Prior to DLS measurements, DMPC MLVs downsized to 100 nm using an extruder. (**b**) Relationship between surface tension and concentration of SF in PBS buffer (pH 7.4, 25 °C). Each white circle represents an individual measurement with dashed line as a guide to the eye.

The concentration-dependent variations in the average diameter of the DMPC/SF (=1/1) mixed solutions showed that the average diameters remained relatively constant below 10^−^^5^ M, gradually decreasing as concentrations surpassed 10^−5^ M (Figure 3a). The concentration was highly consistent with the critical aggregation concentration (CAC) of SF in PBS (1.3 × 10^−5^ M), as shown in Figure 3b. When SF is adsorbed at the air–water interface, the alkyl chain is directed out of water and the cyclicpeptide moiety is directed into water. Previously, we have reported that an SF monolayer exhibited a liquid expanded monolayer at the air–water interface, demonstrating that the SF is in a loose molecular packing state owing to its bulky heptapeptide ring [24]. When the interface is completely occupied by SF, the excess molecules of SF start to aggregate into micelles. Indeed, the SEC analysis of an SF solution above CAC shows the formation of micelles whose diameter was estimated to be 6.8 nm, as shown in Figure 1b. Thus, above the CAC, SF micelles solubilize MLVs. Thus far, the results clearly demonstrate that nanodisc formation is induced in the presence of SF above its CAC: SF adsorbs at the air–water interface at low concentrations, and after reaching its adsorption equilibrium, it begins to form self-associates in aqueous solution. 

Consequently, we concluded that the DMPC/SF complex spontaneously assembles into nanodiscs in aqueous solutions. The interaction between SF and biomimetic membrane models has been previously reported: atomic-force microscopy imaging on lipid monolayers confirmed the formation of SF-rich clusters upon mixing SF with DMPC [25,26]. This phase separation phenomenon is associated with the strong interactions between SF molecules. It is comparable to their association with DMPC, possibly originating from a hydrophobic mismatch and ultimately promoting the immiscibility of the two components. Consequently, the structure of the nanodiscs observed in our study can be explained by the formation of discoidal aggregates when SF envelops the circumference of a DMPC bilayer, adopting a belt-like configuration.

**Figure 4:**
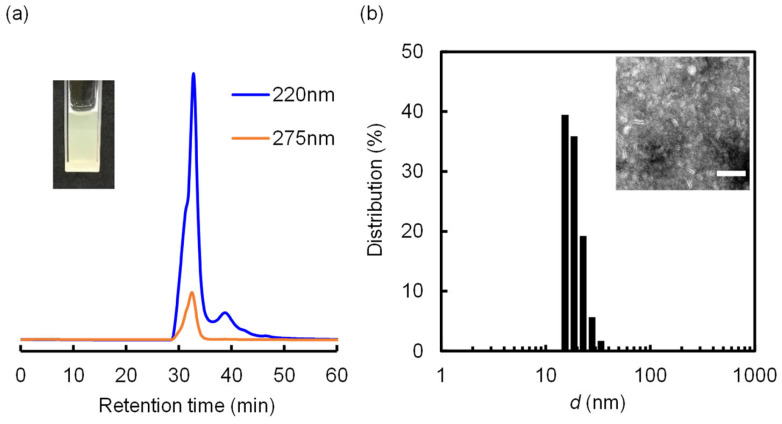
(**a**) SEC profiles of the nanodiscs prepared from SF, DMPC, and CoQ10 at 25 °C. The inset shows visual observation of the sample. (**b**) DLS size distribution of the nanodiscs prepared from SF, DMPC, and CoQ10. The inset shows an NS-TEM image of the nanodiscs prepared from SF, DMPC, and CoQ10 (scale bar = 50 nm).

The nanodiscs (SF = DMPC = 1 mM) can incorporate CoQ10, a strongly hydrophobic lipid that functions as an antioxidant [27,28]. Notably, UV/vis spectroscopy of CoQ10-incorporated nanodiscs revealed a characteristic absorbance peak at 275 nm. The SEC analysis of the CoQ10-incorporated nanodiscs gave rise to major absorbance peaks at 275 and 220 nm corresponding to CoQ10 and SF, respectively (Figure 4a). This spectral pattern suggests that CoQ10 is embedded within the inner region of the nanodiscs. DLS measurement revealed a narrow size distribution, with an average diameter of 18.0 ± 3.6 nm (Figure 4b). The NS-TEM image clearly shows circular and rod-like objects corresponding to the top and side views of the nanodiscs. In essence, a facile method to solubilize significant quantities of CoQ10 in nanodisc was developed herein.

### 2.3. Phase Diagram

**Figure 5:**
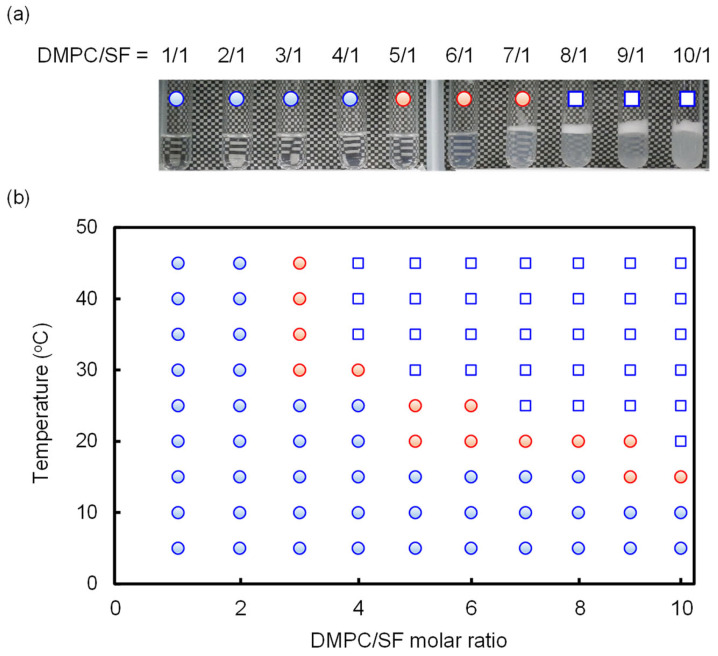
(**a**) Visual observation of the solutions of DMPC/SF mixtures at different molar ratios (25 °C). (**b**) Phase diagram of DMPC/SF mixture at different temperatures. Initial concentrations of SF were all set to 20 mM. Each blue circles represent a transparent solution of low viscosity, the blue squares represent turbid solutions of low viscosity, and the red circles represent transparent solutions of high viscosity.

**Figure 6:**
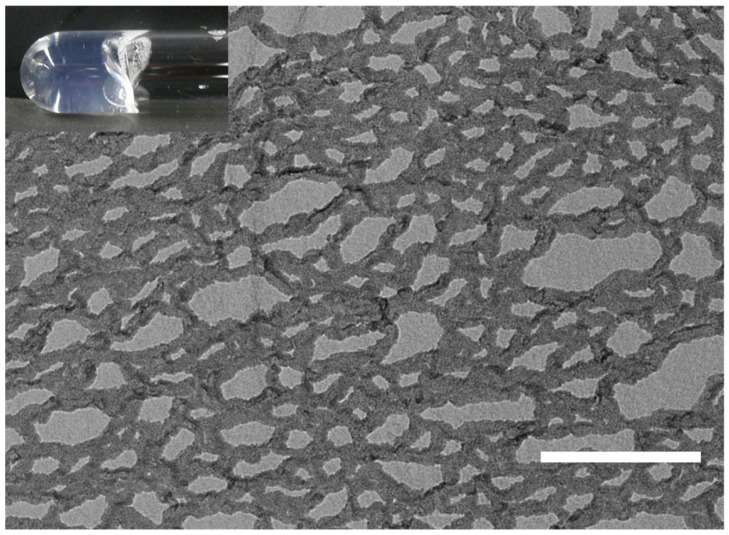
TEM image of a hydrogel prepared using the freeze-etching technique (SF = 20 mM; DMPC = 100 mM in a PBS buffer at pH 7.4; scale bar = 200 nm). The inset shows a photograph of the hydrogel sample.

This study investigated variations in the solubility of mixed DMPC/SF solutions with varying molar ratios (DMPC/SF = 1/1, 2/1, 3/1, 4/1, 5/1, 6/1, 7/1, 8/1, 9/1, and 10/1) at 25 °C (initial concentrations of SF were all set to 20 mM), and the summarized results are presented in Figure 5a. SF demonstrated the capacity to solubilize up to 5 equiv. of DMPC, yielding transparent solutions of low viscosity. The phase diagram of the DMPC/SF mixture at different temperatures is summarized in Figure 5b. Notably, this diagram clearly delineates the solubility threshold of the DMPC/SF mixture; the solubility increases with decreasing temperature, and SF can solubilize 10 equiv. of DMPC at 5 °C. Remarkably, the phase diagram also revealed that the viscosity of the DMPC/SF mixture increased significantly around the border between the transparent and turbid solutions, as indicated by the red circles. The TEM image of the hydrogel (SF = 20 mM, DMPC = 100 mM at 25 °C) shows fibrous structures entangled with each other as shown in Figure 6. It is well known that SF can adopt several conformations because of the flexibility of the cyclic peptide moiety. Therefore, the self-assembled structures of SF vary significantly depending on the external conditions. Akiba et al. reported that cyclohexane solutions of SF form organogels by entanglement of supramolecular nanofibers [29]. Ikeda et al. reported a phospholipid-specific formation of lipid–peptide nanofibers [30]. These results suggest that the fibrous structures, as shown in Figure 6, of the DMPC/SF mixture consist of a single structural unit assembly. 

## 3. Materials and Methods

### 3.1. Materials 

SF and CoQ10 were purchased from Kaneka Corporation (Osaka, Japan) and used without further purification. l-α-dimyristoylphosphatidylcholine (DMPC, 99.0%) was purchased from NOF Corporation (Tokyo, Japan) and was used as received. Phosphate buffer powder (FUJIFILM Wako Chemicals, Osaka, Japan) was used for preparation of PBS buffer solution (67 mM phosphate, pH 7.4).

### 3.2. SF−DMPC Nanodisc Preparation [16]

DMPC was dissolved in chloroform in a test tube. The solvent was then removed by blowing nitrogen gas into the test tube. The test tube was stored overnight at room temperature under reduced pressure to give a thin lipid film on the test tube wall. PBS buffer was then added to the film, and the test tube was shaken vigorously on a vortex mixer to give the MLV aqueous solution. The formation of MLVs was confirmed by using an optical microscope. We performed the direct conversion from a MLV to a nanodisc by adding SF solution, vortexing the sample for 3 min, and subsequently incubating the sample for 12 h at 25 °C [12]. 

### 3.3. Surface Tension Measurement

The surface tension measurement was performed by the pendant drop method at 25 °C, using an apparatus consisting of an automatic interfacial tensiometer (DM500, Kyowa Interface Science, Saitama, Japan) and the Drop Shape Analysis software of FAMAS version 2.01. The tip of the syringe was used for preparation of a drop. A drop profile was extracted from the drop image. A curve-fitting program compared the experimental drop profile with a theoretical one (the Young−Laplace method) and gave the corresponding surface tension value [12]. The concentration of SF stock solution in PBS buffer was diluted to each concentration. The critical aggregation concentration (CAC) and surface tension at the CAC (γ_CMC_) were determined from the curve of the break point of surface tension versus the logarithm of the concentration.

### 3.4. Size-Exclusion Chromatography (SEC)

First, samples were passed through a PVDF filter (0.45 μm) and injected onto a Superose 6 10/300 GL column (GE Healthcare, Chicago, IL, USA) at 25 °C at a flow rate of 0.5 mL min^−1^ using an AKTA prime plus chromatography system (GE Healthcare, Chicago, IL, USA). Phosphate buffer (67 mM phosphate, pH 7.4) was used as a running buffer. The void volume (8.3 mL) was confirmed by injecting MLVs downsized to 100 nm using an extruder. The standards were thyroglobulin (669,000 Da, 17 nm), ferritin (440,000 Da, 12.2 nm), aldolase (158,000 Da, 9.6 nm), ovalbumin (44,000 Da, 6.1 nm), and ribonuclease A (13,700 Da, 3.3 nm), which gave retention volumes of 13.3, 15.3, 16.6, 17.7, and 19.0 mL, respectively.

### 3.5. Dynamic Light Scattering (DLS) 

The size distributions were measured using a DLS instrument (DLS-7000, Otsuka Electronics Co., Osaka, Japan) using a 488 nm Ar laser (75 mW) as a light source (25 °C). The time-dependent correlation function of the scattered light intensity was measured at a scattering angle of 90°. The particle size distributions were determined using the software provided with the instrument and the autocorrelation function was analysed using the histogram method.

### 3.6. Transmission Electron Microscopy (TEM)

#### 3.6.1. Negative Staining

For the nanodiscs, negative-stein TEM (NS-TEM) measurements were conducted. A glow-discharged copper grid coated with carbon (Excel support film, 200 mesh, Nisshin EM Co., Tokyo, Japan) was immediately inverted onto a 2 μL droplet of the sample solution placed on Parafilm. After 1 min, the sample solution was blotted using filter paper, and the grid surface was placed in contact with 20 μL water. After blotting water with filter paper, the grid surface was treated again with a second drop of water. 

In this work, we used phosphor tungstic acid and uranyl acetate as stains. The grid was immediately placed onto a 25 μL droplet of aqueous 1% phosphor tungstic acid or uranyl acetate. After 30 s, the excess stains were removed and the grid was allowed to dry thoroughly. In the case of phosphor tungstic acid, images were taken on an H-7650 transmission electron microscope (Hitachi High-Technologies, Tokyo, Japan) at 120 kV. In the case of uranyl acetate, images were taken on a JEM-1400 Plus transmission electron microscope (JEOL, Tokyo, Japan) at 120 kV.

#### 3.6.2. Freeze Etching 

The nanostructure of SF/DMPC hydrogel was analysed by quickly immersing the fully swollen hydrogel sample in slush nitrogen. The etching process was performed with a specimen preparation system (JSF-V, JEOL, Tokyo, Japan). The sample was cut using a microtome blade and, subsequently, sublimation was performed to visualize the structure clearly. Then, the fractured surface was replicated by first evaporating platinum and then carbon to strengthen the replica. After the replica was washed, it was examined and photographed using a transmission electron microscope (JEM-1010, JEOL, Tokyo, Japan) [31].

## 4. Conclusions

This paper reports the use of SF as a pivotal molecular component for nanodiscs having enhanced membrane scaffolding properties. Our results highlight the feasibility of preparing nanodiscs without the need for laborious procedures, thereby showcasing their potential to serve as promising carriers for various organic compounds such as CoQ10. The lipid bilayer nanodiscs consisting of SF as a bio-derived amphiphile are considered to be inherently biocompatible because they mimic the nature of nanodiscs produced in our bodies. Thus, nanodiscs prepared from SF and DMPC is expected to be not only useful platforms for the study of membrane proteins but also biocompatible drug carriers. Additionally, the regulated self-assembly of the DMPC/SF mixture, leading to the formation of fibrous architectures, holds the potential to function as theranostic nanomedicine. Further studies on the incorporation of membrane proteins and biocompatible drugs within nanodiscs are currently underway in our laboratory.

## Data Availability

The data presented in this study are available on request from the corresponding author.
